# Genomic regulation of natural variation in cortical and noncortical brain volume

**DOI:** 10.1186/1471-2202-7-16

**Published:** 2006-02-17

**Authors:** Jackson Beatty, Rick E Laughlin

**Affiliations:** 1 Department of Psychology, University of California Los Angeles, Los Angeles, California, USA

## Abstract

**Background:**

The relative growth of the neocortex parallels the emergence of complex cognitive functions across species. To determine the regions of the mammalian genome responsible for natural variations in cortical volume, we conducted a complex trait analysis using 34 strains of recombinant inbred (Rl) strains of mice (BXD), as well as their two parental strains (C57BL/6J and DBA/2J). We measured both neocortical volume and total brain volume in 155 coronally sectioned mouse brains that were Nissl stained and embedded in celloidin. After correction for shrinkage, the measured cortical and noncortical brain volumes were entered into a multiple regression analysis, which removed the effects of body size and age from the measurements. Marker regression and interval mapping were computed using WebQTL.

**Results:**

An ANOVA revealed that more than half of the variance of these regressed phenotypes is genetically determined. We then identified the regions of the genome regulating this heritability. We located genomic regions in which a linkage disequilibrium was present using WebQTL as both a mapping engine and genomic database. For neocortex, we found a genome-wide significant quantitative trait locus (QTL) on chromosome 11 (marker D11Mit19), as well as a suggestive QTL on chromosome 16 (marker D16Mit100). In contrast, for noncortex the effect of chromosome 11 was markedly reduced, and a significant QTL appeared on chromosome 19 (D19Mit22).

**Conclusion:**

This classic pattern of double dissociation argues strongly for different genetic factors regulating relative cortical size, as opposed to brain volume more generally. It is likely, however, that the effects of proximal chromosome 11 extend beyond the neocortex strictly defined. An analysis of single nucleotide polymorphisms in these regions indicated that ciliary neurotrophic factor (*Cntf*) is quite possibly the gene underlying the noncortical QTL. Evidence for a candidate gene modulating neocortical volume was much weaker, but *Otx*1 deserves further consideration.

## Background

### Cortex and cognition

The absolute and relative volumes of anatomically defined brain regions-such as the mammalian cerebral cortex-are of functional importance both within and across species [1-4]. In humans, the volume of the cerebral hemispheres ranges between 850 and 1380 cm^3 ^in young adults [5]. Further, neocortical size is hereditarily specified, with over eighty percent of the variance of human neocortical gray matter volume being genetically determined [6]. At present, little is known about the genomic determinants of such natural variation.

Further, cognitive ability is related to neocortical size. For example, Reiss et al. showed that IQ is positively correlated with cerebral volume in children [7]. Thompson et al. provided evidence based on quantitative MRI volumetric measurements that not only is neocortical volume genetically determined (*h*^2 ^> 0.8), but that Spearman's *g*, a measure of fluid intelligence, was significantly linked to frontal lobe neocortical volume. Similar findings also have been reported by Posthuma et al. [9].

These results provide evidence that neocortical volume is very much genetically determined and linked to cognitive abilities. However, such studies provide no evidence concerning the genomic mechanisms that underlie these highly heritable traits. For this all important question, quantitative neuroanatomical studies of the neocortex of recombinant inbred strains of mice provide one important path to unravelling the genomics of brain size. The discovery of the genes that differentially regulate neocortical volume is a primary question for contemporary cognitive neuroscience. The study of RI mice might provide some insight into this problem. Here, we report the first empirical study of this fundamental problem.

### Experimental strategy

We measured neocortex and total brain volume in 155 mice from 34 RI strains (BXD) as well as their two parental strains, C57BL/6J (B) and DBA/2J (D), all strains being homozygous throughout their genomes. From these measurements, both in vivo cortical brain and in vivo noncortical brain volumes were calculated.

## Results

### Reliability of measurement

To assess the reliability of the stereological measurements, cortical brain area was remeasured blindly in 94 brain sections. The test-retest reliability coefficient indicated that the measurements were highly reliable (*r *= 0.984). Similarly, reliability for total fixed brain volume remeasured for twenty mice was also very high (*r *= 0.996).

### Regression analyses

The size of brain structure is not only regulated by structure-specific genes, but varies with other factors, which may include body weight (BW), age, and sex. To statistically remove these influences from our histological phenotypes, a multiple-regression analysis was performed using body weight, the logarithm of age, and sex as predictor variables, a standard procedure in QTL analysis. Body weight and the logarithm of age were the only significant predictor variables for in vivo cortical brain volume, yielding the following regression equation for adjusted cortical volume:

*C*_*x*_*V*_*Reg *_= 111.68 + 1.16(*BW*) - 18.38(ln(*Age*)),

henceforth referred to as *neocortex*.

The same two predictor variables were used for the regression of in vivo noncortical brain volume, although body weight was the only significant predictor in that analysis. The regression equation for in vivo noncortical brain volume was:

*NC*_*x*_*V*_*Reg *_= 270.53 + 3.87(*BW*) - 17.86(ln(*Age*)),

termed *noncortex *in the following analyses.

### QTL mapping

The linkage disequilibrium between a measured phenotypic trait, like regressed cortical volume or regressed noncortical volume, and any genomic marker can be measured using the likelihood ratio statistic (LRS). This is a quantitative estimate of the probability that each individual marker does or does not overlie one of the polygenes regulating the phenotypic trait.

We computed the LRS for each of 3,795 marker positions on chromosomes 1 through 19 as well as chromosome X, using webQTL as a genotype source and mapping engine [10,11]. Similar results were also obtain in preliminary analyses using the previous marker set containing 788 more sparsely distributed WebQTL markers.

The LRS depends upon the ratio of a model not incorporating information about the marker in question (the *reduced model*) to a model incorporating genetic information (the *full model*), as shown in Equation 3. In marker regression, the LRS for a particular marker is a function of the ratio of the sum of squared deviations from the mean for the phenotypic variable *x *to the sum of the squared deviations of the variable from the submeans that are obtained when the phenotypes are segregated according to the parental allele value at that marker:

LRS=Nln⁡SSReducedModelSSFullModel=Nln⁡∑(xA−x¯A)2∑(xB−x¯B)2+∑(xD−x¯D)2
 MathType@MTEF@5@5@+=feaafiart1ev1aaatCvAUfKttLearuWrP9MDH5MBPbIqV92AaeXatLxBI9gBaebbnrfifHhDYfgasaacH8akY=wiFfYdH8Gipec8Eeeu0xXdbba9frFj0=OqFfea0dXdd9vqai=hGuQ8kuc9pgc9s8qqaq=dirpe0xb9q8qiLsFr0=vr0=vr0dc8meaabaqaciaacaGaaeqabaqabeGadaaakeaafaqadeGadaaabaGaemitaWKaemOuaiLaem4uamfabaGaeyypa0dabaGaemOta4KagiiBaWMaeiOBa42aaSaaaeaacqWGtbWucqWGtbWudaWgaaWcbaacbiGae8NuaiLae8xzauMaemizaqMaemyDauNaem4yamMaemyzauMaemizaqMaemyta0Kaem4Ba8MaemizaqMaemyzauMaemiBaWgabeaaaOqaaiabdofatjabdofatnaaBaaaleaacqWGgbGrcqWG1bqDcqWGSbaBcqWGSbaBcqWGnbqtcqWGVbWBcqWGKbazcqWGLbqzcqWGSbaBaeqaaaaaaOqaaaqaaiabg2da9aqaaiabd6eaojGbcYgaSjabc6gaUnaalaaabaWaaabqaeaaieaacqGFOaakcqWF4baEdaWgaaWcbaGae8xqaeeabeaakiabgkHiTiqbdIha4zaaraWaaSbaaSqaaiabdgeabbqabaGccqGGPaqkdaahaaWcbeqaaiabikdaYaaaaeqabeqdcqGHris5aaGcbaWaaabqaeaacqGGOaakcqWG4baEdaWgaaWcbaGaemOqaieabeaakiabgkHiTiqbdIha4zaaraWaaSbaaSqaaiabdkeacbqabaGccqGGPaqkdaahaaWcbeqaaiabikdaYaaakiabgUcaRmaaqaeabaGaeiikaGIaemiEaG3aaSbaaSqaaiabdseaebqabaGccqGHsislcuWG4baEgaqeamaaBaaaleaacqWGebaraeqaaaqabeqaniabggHiLdGccqGGPaqkdaahaaWcbeqaaiabikdaYaaaaeqabeqdcqGHris5aaaaaaaaaa@7E06@

where the subscripts A, B, and D indicate, respectively, all strains, strains with the C57BL/6J B-type, and strains with the DBA/2J D-type allele at the marker, as shown above. N is the total number of strains analyzed, here 36. The value of the LRS can be compared with values obtained from a permutation test to determine the genome-wide significance of the association [12].

### QTL boundaries

The boundaries of each QTL were determined by examination of the distribution of the LRS scores of the markers surrounding the QTL peak. All peaks were well formed and sharp. Following Van Ooijen [13] we located the nearest markers for which the value of the LRS was reduced by at least 9.2 units, corresponding to the 95 percent confidence limits for the QTL. Kim et al. has recently confirmed that this method is both accurate and unbiased [14].

### Descriptive statistics

There was substantial variation in both in vivo neocortical and noncortical brain volume across strains. In vivo neocortical volume ranged from a minimum of 77.3 mm^3 ^in BXD30 mice to 125.1 mm^3 ^in BXD5 mice.

The mean in vivo neocortical volume was 101.9 mm^3^, with a standard deviation of 9.8. In vivo noncortical brain volume ranged from 266.6 (BXD27) to 391.7 mm^3 ^(BXD5), with a mean of 319.9 and a standard deviation of 25.2. The range of both phenotypic measurements is about 45 percent of the mean phenotypic value, which provides ample variation for a linkage disequilibrium analysis.

### Analysis of variance

The hypothesis that neocortical volume is in part genomically determined was assessed by analysis of variance (ANOVA), with inbred strain (both parental and recombinant) as the independent variable and residual neocortical volume as the dependent variable, as shown in Table 2. The main effect of strain was highly significant (*F*_35,119 _= 5.909, *p *< .0001). Significant strain differences were also observed for residual noncortical brain volume (*F*_35,119 _= 8.726, *p *< .0001).

### Relative independence of cortical volume and body weight

Brain size is known to vary with body size in a wide variety of species, including mice and other rodents [3,15]. However, an examination of the regression analyses reported above for phenotype versus predictor variables suggests that the covariation between brain volume and body size is very much weaker for cerebral cortex than it is for noncortical brain volume. We tested this relationship by first computing the Pearson product moment correlation coefficients for body weight and in vivo cortical volume (*r *= .224) and in vivo noncortical volume (*r *= .537). We then used Fisher's *r*-to-*z *transformation [[16], page 49] to statistically test for a significant difference between these correlations. The result provides evidence that the mammalian cerebral cortex is significantly less regulated by factors related to body size than are most other regions of the central nervous system (*z *= -3.235, *p *< 0.01).

### High heritability of brain size

The proportion of phenotypic variance that might be attributed to genomic factors was estimated as broad-sense heritability, or the total genetic determination of a phenotypic trait. Broad-sense heritability was estimated from an analysis of variance with strain as the independent variable and quantitative phenotype as the dependent variable, using the following formula:

ω2=F−1F+(dfw+1)/dfb,
 MathType@MTEF@5@5@+=feaafiart1ev1aaatCvAUfKttLearuWrP9MDH5MBPbIqV92AaeXatLxBI9gBaebbnrfifHhDYfgasaacH8akY=wiFfYdH8Gipec8Eeeu0xXdbba9frFj0=OqFfea0dXdd9vqai=hGuQ8kuc9pgc9s8qqaq=dirpe0xb9q8qiLsFr0=vr0=vr0dc8meaabaqaciaacaGaaeqabaqabeGadaaakeaaiiGacqWFjpWDdaahaaWcbeqaaiabikdaYaaakiabg2da9maalaaabaGaemOrayKaeyOeI0IaeGymaedabaGaemOrayKaey4kaSIaeiikaGIaemizaqMaemOzay2aaSbaaSqaaiabdEha3bqabaGccqGHRaWkcqaIXaqmcqGGPaqkcqGGVaWlcqWGKbazcqWGMbGzdaWgaaWcbaGaemOyaigabeaakiabcYcaSaaaaaa@436E@

where *F *is the ratio of the mean square deviation between strains to the mean square deviation within strains. The value of *ω*^2 ^varies between 0.0 and 1.0. These analyses showed that a substantial fraction of phenotypic variance is genetically determined. For regressed cortical volume, *ω*^2 ^was 0.53; for regressed noncortical volume, *ω*^2 ^was 0.64.

### QTLs for cortical volume

Given the high heritability of both residual cortical and noncortical brain volume, the genomic question is not *if *quantitative trait loci exist for these phenotypic anatomical variables (certainly they must exist or heritability could not be large), but rather *where *on the genome these QTLs are located.

**Figure 1:**
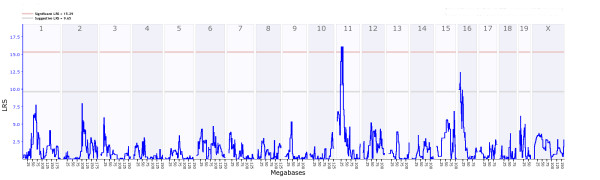
**QTL Plot for Residual Neocortal Volume**. The red upper line indicates the LRS value for genome-wide significant QTL and the lower grey line the level of suggestive QTL according to the criteria of Lander and Kruglyak [33]. There is a genome-wide significant linkage disequilibrium located in the proximal portion of chromosome 11. There is also a suggestive association on proximal chromosome 16. Notice that there is no significant association between neocortical volume and any marker on chromosome 19.

QTL analyses provide the mechanism for locating genomic regions in which allele values are closely associated with a phenotypic trait. The results of the QTL analysis of 3,795 markers across the mouse genome for association with regressed cortical volume are shown in Figure 1. There are two prominent spikes in the LRS profile for residual cortical volume. Table 3 shows the LRS values produced by marker regression.

The most significant marker (p ≤ 0.05 genome-wide) is D11Mit19 at 25.4 megabases (mb) on chromosome 11. Two other nearby markers (S11Gnd020.100 and D11Mit135) also show suggestive association with regressed cortical volume. Because of the restricted genomic resolution afforded by the available number of recombinant inbred strains, we treat these three markers as reflective of a single QTL in the immediate vicinity of D11Mit19.

There is a second, suggestive QTL for residual cortical volume on chromosome 16. This is reflected by a linkage disequilibrium with two adjacent markers, D16Mit9 (6.0 mb) and D16Mit100 (11.5 mb). These markers on proximal chromosome 16 also contribute to the genetic specification of neocortical volume.

To estimate the minimal proportion of phenotypic variance accounted for by each of these two QTL markers, we calculated the partial eta squared (ηp2
 MathType@MTEF@5@5@+=feaafiart1ev1aaatCvAUfKttLearuWrP9MDH5MBPbIqV92AaeXatLxBI9gBaebbnrfifHhDYfgasaacH8akY=wiFfYdH8Gipec8Eeeu0xXdbba9frFj0=OqFfea0dXdd9vqai=hGuQ8kuc9pgc9s8qqaq=dirpe0xb9q8qiLsFr0=vr0=vr0dc8meaabaqaciaacaGaaeqabaqabeGadaaakeaaiiGacqWF3oaAdaqhaaWcbaGaemiCaahabaGaeGOmaidaaaaa@30E7@) from an analysis of variance with the markers D11Mit19 and D16Mit100 and their interaction as factors. These proportions are 0.22 for D11Mit19 and 0.14 for D16Mit100. The proportion attributable to the interaction of the two markers was negligible.

### QTLs for noncortical brain volume

**Figure 2:**
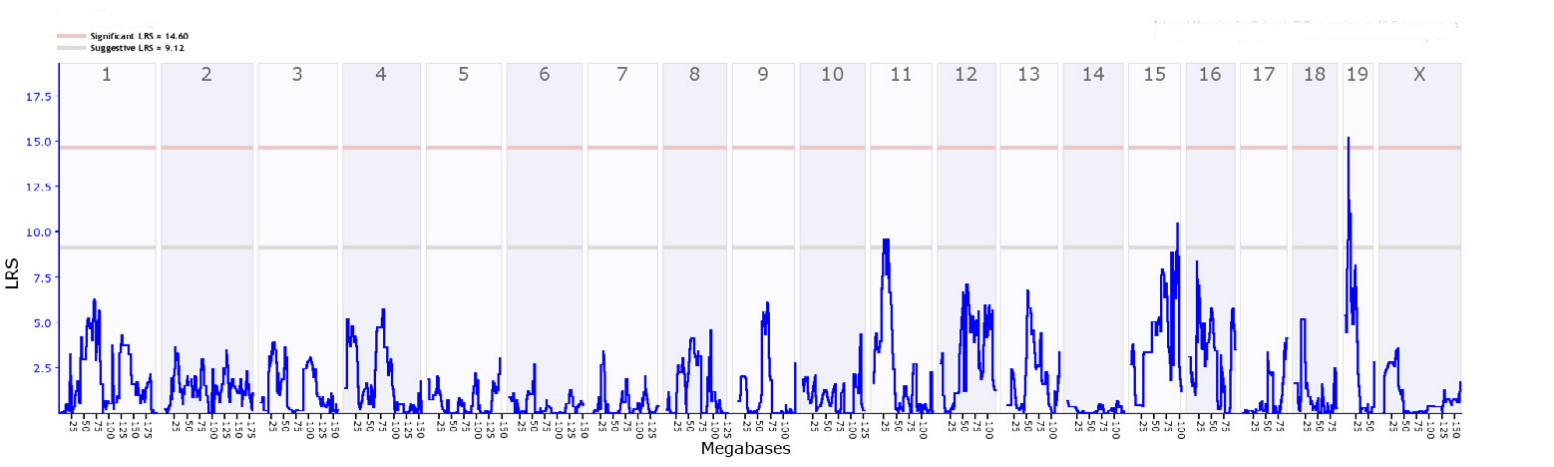
**QTL Plot for Residual Noncortal Volume**. Here, the effect of the QTL on proximal chromosome 11 is attenuated and no longer significant. There is a suggestive association on the distal portion of chromosome 15 and a significant QTL on chromosome 19. Thus, there is a double dissociation between neocortex and noncortex for significant QTLs on chromosomes 11 and 19.

There are significant and suggestive QTLs for regressed noncortical brain volume as well, two of which do not affect cortical volume, and the third affects both, but to differing degrees. Figure 2 presents the corresponding QTL analysis for regressed noncortical brain volume.

A significant association between this regressed noncortical volume and the mammalian genotype may be seen on proximal chromosome 19. This is most evident at marker D19Mit22 (10.0 mb), which reaches genome-wide significance on many – but not all – permutation tests. Because the results of all permutation tests vary slightly from sample to sample, any LRS score that lies within this narrow range will reach significance with some instances of the permutation test, but not others. Thus, we conclued that this QTL neither greatly exceeds nor falls far short of the genome-wide 0.05 significance level.

This QTL extends to neighboring markers D19Mit42 (9.4 mb) and D19Mit127 (12.1 mb). These markers appear to be indicative of a single QTL harboring one or more genes that regulate noncortical, but not cortical, brain size.

D11Mit19 on Chromosome 11, which is significantly related to regressed cortical volume, also shows a suggestive, but not significant, association with noncortical brain volume.

There is also a marker on chromosome 15 with a borderline LRS in marker regression, D15MIT72 (84.5 mb), which exceeds the criterion for a suggestive LRS on some permutation tests, but not on others.

The minimal proportion of phenotypic variance accounted for by these three markers was estimated by ηp2
 MathType@MTEF@5@5@+=feaafiart1ev1aaatCvAUfKttLearuWrP9MDH5MBPbIqV92AaeXatLxBI9gBaebbnrfifHhDYfgasaacH8akY=wiFfYdH8Gipec8Eeeu0xXdbba9frFj0=OqFfea0dXdd9vqai=hGuQ8kuc9pgc9s8qqaq=dirpe0xb9q8qiLsFr0=vr0=vr0dc8meaabaqaciaacaGaaeqabaqabeGadaaakeaaiiGacqWF3oaAdaqhaaWcbaGaemiCaahabaGaeGOmaidaaaaa@30E7@. The obtained values were 0.25 for D19Mit22, 0.13 for D11Mit19, and 0.13 for D15Mit72. All interactions produced negligible values of ηp2
 MathType@MTEF@5@5@+=feaafiart1ev1aaatCvAUfKttLearuWrP9MDH5MBPbIqV92AaeXatLxBI9gBaebbnrfifHhDYfgasaacH8akY=wiFfYdH8Gipec8Eeeu0xXdbba9frFj0=OqFfea0dXdd9vqai=hGuQ8kuc9pgc9s8qqaq=dirpe0xb9q8qiLsFr0=vr0=vr0dc8meaabaqaciaacaGaaeqabaqabeGadaaakeaaiiGacqWF3oaAdaqhaaWcbaGaemiCaahabaGaeGOmaidaaaaa@30E7@.

### Composite interval mapping

Composite interval mapping [17] can increase the sensitivity of a search for additional QTLs associated with a phenotype by incorporating the variance associated with previously identified significant QTLs to the background of the additive model. Composite interval maps were constructed for both residual phenotypes, but no additional QTLs emerged from these analyses.

### Epistasis

Epistasis is the interaction of two or more genes at different chromosomal sites, which can either be suppressive or facilitative. It is important to test for epistasis, as it indicates a departure from additivity in polygene action.

We tested for epistasis between all the possible marker pairs for the regressed neocortical and noncortical volume phenotypes, respectively, using Ljungberg's DIRECT global optimization algorithm as implemented in WebQTL with phenotype data from the recombinant inbred strains [18]. There were no significant interactions for either phenotypic variable.

## Discussion

These results have several implications for understanding the genetic specification of neocortical development.

### Relative independence of cortical size from body size

The various strains of BXD mice differ considerably from each other in both cortical and noncortical brain volume. However, these two compartments of the mouse brain show quite different dependencies on body size. Nearly 30 percent of the variance in noncortical brain volume can be accounted for by body size in our data (see Striedter [19] for a history of the study of brain and body size).

In contrast, our measurements suggest that neocortical brain volume is much less dependent on body size, with only about 5 percent of that compartment being predictable from body weight. Neocortex appears to be regulated by factors that differ substantially from those regulating other regions of the central nervous system, a conclusion that complements recent observations made by Bush and Allman [4].

Thus, one might expect different genomic regions to regulate the heritable factors that determine size of the neocortex and noncortical brain regions, which proves to be the case.

### A cortex-dependent QTL on chromosome 11

One QTL with genome-wide significance for regressed cortical volume emerged from our analyses. It was located at the DNA marker D11Mit19 and flanked by two suggestive markers.

A QTL associated near this location (marker D11Mit53 at 16.0 centiMorgans) was reported previously in a doctoral dissertation in which the phenotype was total brain weight, controlling for the effects of sex, age, body weight, litter size, and parity by multiple regression [20]. The QTL was interpreted as one that controls neuron number throughout the brain.

Our data suggest a more specific interpretation. Using our phenotypic data we can compare the the proportion of variance accounted for *R*^2 ^on chromosome 11 at D11Mit19 for neocortex alone (0.34), whole brain volume or brain weight (0.29), and noncortex (0.21), all regressed for body weight and the logarithm of age. The *R*^2 ^values are highest for neocortex, lowest for noncortex, and intermediate for the whole brain. It is clear that this QTL is not a generic determinant of brain size, but rather selectively exerts its effects on the neocortex and perhaps some adjacent forebrain structures such as the olfactory bulb.

### Candidate genes for a cerebral cortex volume modulator

The QTL analyses presented above describe chromosomal regions in which a phenotypic measurement – here cortical or noncortical brain volume – are statistically linked with chromosomal markers. But the true endgame of neurogenomics is the discovery of specific genes that mediate these associations. We describe three approaches for narrowing this search.

The mouse chromosome 11 is slightly less than 123 megabases (mb) in length. The strongest linkage between cortical volume and any marker in the murine genome is at D11Mit19 at 25.5 mbp on chromosome 11. The ± 95% confidence intervals for this QTL are between 20 and 35 mb.

Using the criteria described above, SNP filtering revealed polymorphisms in the coding or untranslated regulatory regions of twenty known genes. The filtered known genes within the chromosome 11 confidence interval for neocortex are E030025D05Rik, AW011752, 4931428D14Rik, C330012F17Rik, 2010316F05Rik, 2510006C20Rik, *Stc2, Il9r*, 3300001G02Rik, *Rhbdf1, Mpg, Hba-x*, A230090E05, *Stk10, Fbxw1b, Fgf18, Gabrp, Kcnmb1, Dock2*, and *Vrk2*. However, a detailed examination of these genes suggests that they are not uniquely expressed in brain and thus are less likely to provide an explanation of the neocortex QTL.

Nonetheless, several possibilities remain. First, and most obvious, is that the gene(s) controlling neocortical volume have simply not yet been discovered, a possibility that can not be excluded, particularly since the study of the genome is still young.

Another possibility is that the gene may have been "discovered" but its effects on the developing forebrain are not yet known. In this case, the known gene would not be selected in the literature search.

A third possibility is that the gene *is *known, but was excluded in SNP filtering. Thus, it is of interest that there is a known gene within the QTL on chromosome 11 that is specifically related to neocortex development. *Otx1 *(chromosome 11 at 21.8 mb) plays a major and selective role in the development of the mammalian neocortex [21,22]. *Otx1 *is expressed early in development and provides a molecular basis for the development of the deep layers of the cerebral cortex, as well as in the development of the eye, olfactory system, and cochlea.

*Otx1 *is closely related to *Otx2*, which modulates the development of the mammalian brainstem. Interestingly, the expression of *Otx2 *is regulated by two distant enhancers, one located 75 kb upstream and the other 115 kb downstream from *Otx2 *itself on chromosome 14 [23]. If similar enhancers exist for *Otx1*, they would have been excluded by our SNP filtering procedure. A search of the Celera database reveals that there are 85 D/B SNPs within ± 150 Kb of Otx1. If any of these SNPs lay within a putative Otx1 enhancer, it could provide a molecular basis for the QTL. Thus, *Otx1 *may still be a reasonable candidate gene mediating the effects of the chromosome 11 QTL for neocortical volume.

### A noncortex-dependent QTL on chromosome 19

In the analysis of regressed noncortical volume, the single QTL detected with genome-wide significance was on proximal chromosome 19. This genomic region had absolutely no effect on regressed cortical volume. Thus, the data display a classical pattern of double dissociation: one factor that affects cortical volume more than noncortical volume and a second factor that shows the mirror image pattern. Double dissociation is an important criterion in neuropsychology, because it provides positive logical proof of the specificity of effects. The same argument can be made in the present case as well.

### Candidate genes for a noncortical volume modulator

In an analysis similar to that performed for neocortex we carried out a SNP filtered Celera search of chromosome 19, which is about 60 mb in length, in the vicinity of D19Mit22, using a QTL confidence interval 13 to 40 mb. This search produced 23 known genes with SNPs differentiating the two parental strains. The filtered known genes within the chromosome 19 confidence interval for noncortex are 5730596K20Rik, D630002G06, *Slc22a6, Chrm1, Slc3a2*, 4930563M09Rik, *Gng3, B3Gat3*, 0710001O03Rik, BC021917, 1110006I15Rik, *Zp1, MOR239-5*, A33000E03Rik, *MOR202-8, Keg1, Cntf, MOR202-3, MOR202-15, MOR202-36, MOR266-8, MOR2127-1*, and *MOR212-2*.

Examination of expression information for these 23 known genes in the NCBI Entrez Gene database revealed one gene that was particularly promising as a candidate to mediate the QTL's effect on noncortical brain volume. That gene is ciliary neurotrophic factor (*Ctnf*).

*Ctnf *is a one of the gp130 family of cytokines and acts as a survival factor for a number of different neuronal cell types. Originally discovered as a factor that supports the survival of parasympathetic nerve fibers in the ciliary ganglia, *Ctnf *is now known to play an active role in neuronal maintenance within the central nervous system [24]. Recent reports have also shown that *Ctnf *modulates central nervous system neurogenesis as well [25]. Thus, it is not unreasonable to conclude the genetically controlled natural variations in *Ctnf *expression might modulate brain size in BXD recombinant inbred mice. *Ctnf *is indeed a strong candidate gene for understanding the QTL on chromosome 19 related to noncortical brain volume.

### Other suggestive QTLs

The other suggestive QTLs reported above should be noted, although the evidence for them is not exceptionally strong. These include possible cortex-dependent QTL on proximal 16 and the noncortex dependent QTL on chromosome 15. Suggestive QTLs in today's analyses can provide guidance and support for other, more sensitive, analyses in the future if they are replicated.

## Conclusion

Our localization of QTLs regulating neocortical size may have broader, more cognitive implications. Gibson, in a recent theoretical paper on brain size and mental capacity [[2], p.17], concludes: perhaps "those genetic factors that control enhanced brain size, especially the size of the sensori-motor control for the oral and manual organs, the neocortical association areas, the basal ganglia, and the cerebellum would emerge as the prime genetic determinants of the mental abilities of human infants." Properly designed QTL analyses using the appropriate behavioral phenotypic measures in recombinant inbred mice could help resolve this question.

Since there is ample conservation in the mammalian genome over species, one hopes that findings obtained using well-developed recombinant inbred strains of mice will be applicable to understanding primate and human neurogenomics in a straightforward manner as well.

## Methods

### Mice and specimen preparation

All specimens and associated demographic data were obtained from the Mouse Brain Library or MBL [26,27]. (See  for details.)

All brains were prepared using standard procedures [28]. Each brain was embedded in celloidin and sectioned in 30 micron sections in the coronal plane. Individual sections were stained with cresylechtviolett. Every tenth section was used for measurement, giving a measurement slice thickness of 0.3 mm.

### Histological phenotypes

Histological phenotypes were computed from a set of .3 mm coronal sections by stereological analysis. We defined two raw histological phenotypes, the in vivo cortical volume and in vivo noncortical volume. Demographic data including fresh brain weight, total body weight, age, and sex were obtained for each mouse from the MBL [29].

### In vivo cortical brain volume

In vivo cortical volume was determined for each mouse using the NIH ImageJ digital measurement system. First, the area of the neocortex as it appears in the serial sections was measured by manually tracing the neocortex. Since the boundary of the molecular layer was extremely variable in some images, the dorsal boundary of the neocortex was taken as the inner edge of the molecular layer, which was clearly discernable in all images. The resulting measurements were then corrected for molecular layer area by a factor determined from images in which both the inner and outer boundaries were clear. In the posterior coronal sections, the lateral boundary of the neocortex was taken as the neocortical-piriform cortex border.

Shrinkage during tissue processing is significant and variable, but can be compensated for to produce accurate estimates of in vivo cortical volume. To do so requires measuring the total volume of the processed specimens, which was calculated using ImageJ. The contrast and brightness of all digitized sections were adjusted to permit measurement using the ImageJ particle analysis feature. This automatic feature was highly reproducible. To correct each individual mouse brain for shrinkage, the measured specimen neocortical volume was multiplied by the ratio of fresh brain weight to measured total specimen brain volume, assuming a brain density of about 1.05 mg/mm^3 ^in fixed tissue. This restores the volume lost from the fresh brain during histology. The average volumetric shrinkage was 64%, which corresponds to a linear shrinkage of 29%.

Although there is considerable differential shrinkage *between *specimens, shrinkage is constant across brain structures *within *each individual brain [4].

### In vivo noncortical brain volume

Noncortical brain volume was calculated by first subtracting measured specimen cortical volume from the measured specimen total brain volume, and then correcting for shrinkage in the manner described above. Thus, for each mouse, the sum of its in vivo cortical and noncortical brain volumes equals its fresh brain weight.

### Single Nucleotide Polymorphism (SNP) filtering

We interrogated the Celera Discovery System mouse refSNP database for SNPs within the confidence interval of each QTL following the procedures of Yan (2004). SNPs are single nucleotide polymorphisms, or places on the DNA molecule where different strains of mice exhibit different nucleotides.

Because the BXD mice are recombinant inbred strains arising from C57BL/6J and DBA/2J mice, we filtered the search to include only SNPs that occur between these two parental strains (B-D SNPs). For further analysis, we selected only B-D SNPs that a] occurred within the coding region of the gene as either a mis-match or nonsense mutation, or b] were within the 5'-regulatory region, or c] were present in the 3'-untranslated region. Silent SNPs and SNPs in the intronic or intergenic regions were excluded.

Finally, only SNPs associated with known genes, as determined by a search of the NCBI Entrez Gene database [31], were included. It this way, we were able to identify all known genes within the confidence interval of each QTL.

We also searched for evidence that each of these known genes is expressed morphologically within the brain using Mouse Genome Informatics databases at the Jackson Laboratory [32].

Analyses of these data address a number of issues regarding the genomic determination of neocortical development. These include the natural variation of neocortical and noncortical size in relation to body size, their heritabilities, the localization of the most important genomic regions responsible for both quantities, and identification of known genes that could mediate the observed QTLs.

## Authors' contributions

The intellectual contributions to this paper were shared evenly between the two authors. RL bore primary responsibility for stereological measurement and JB was the principal author of the manuscript.

## Figures and Tables

**Table 1:** Summary of neocortical and related phenotypic measures in 34 BXD recombinant inbred strains and their parental strains. Measured neocortical volume was corrected for the effects of shrinking produced by fixation and embedding in celloidin by multiplying each value by the ratio of the original brain weight to the measured fixed total brain volume. Since the specific gravity of fresh brain is just slightly more than 1.00, brain weight in grams can be taken as a close approximation of brain volume in cubic centimeters. Thus, in vivo brain volume was estimated by fresh brain weight in grams, as provided for each mouse by the Mouse Brain Library. Noncortical volume was estimated as measured total volume minus neocortical volume and was similarly corrected for shrinkage.

**Strain**	**N**	**In Vivo Cortical Volume**	**In Vivo Non-cortical Volume**	**Regressed Cortical Volume**	**Regressed Non-cortical Volume**
C57BL/6J	4	115.59	362.01	123.27	296.84
DBA/2J	4	91.92	293.81	104.63	245.17
BXD1	5	118.47	337.57	126.78	291.27
BXD2	5	99.58	340.76	106.36	274.38
BXD5	5	125.11	391.73	136.07	343.66
BXD6	4	90.41	289.17	103.1	253
BXD8	5	101.54	313.72	111.48	260.54
BXD9	3	107.47	324.06	117.53	278.23
BXD11	3	108.96	311.68	121.34	277.88
BXD12	5	105.06	338.96	114.15	281.95
BXD13	5	103.77	316.63	110.28	259.95
BXD14	5	103.47	340.79	111.53	276.46
BXD15	3	119.99	331.91	128.03	261.79
BXD16	5	115.06	343.66	121.32	285.37
BXD18	4	103.33	317.7	112.84	261.64
BXD19	4	90.66	324.69	101.64	279.25
BXD20	4	97.1	300.73	107.85	254.04
BXD21	4	111.76	320.44	122.58	273.95
BXD22	5	108.19	342.91	116.05	283.27
BXD23	5	100.65	328.09	112.47	283.28
BXD24	4	99.81	310.74	108.36	254.49
BXD25	5	99.65	293.39	113.57	264.54
BXD27	4	93.83	266.62	105.01	214.3
BXD28	4	89	304.6	97.9	255.09
BXD29	4	84.53	295.47	96.48	252.27
BXD30	4	77.29	269.43	90.87	237.01
BXD31	3	95.4	312.87	103.1	252.95
BXD32	3	99.07	357.4	106.77	289.71
BXD33	5	102.47	317.51	110.98	273.87
BXD34	5	101.59	309.03	106.29	249.39
BXD35	4	105.73	313.2	114.86	268.98
BXD36	5	102.7	301.32	113.49	261.82
BXD38	4	98.36	307.59	108.29	266.48
BXD39	5	101.14	307.1	108.94	261.01
BXD40	5	100.47	325.27	111.04	286.65
BXD42	4	101.05	352.17	108.89	303.33

**Table 2:** Effects of Strain on Cortical and Noncortical Volume. The effects of strains (genome) on both cortex and noncortex were analysed by analysis of variance. Both effects were highly significant.

Source	Sum of Squares	df	Mean Square	F	Significance
Strain	12287.62	35	351.07	5.91	.0001
Error	7069.61	119	59.41		
Total	19357.23	154			

**Residual noncortical brain volume:**					

Source	Sum of Squares	df	Mean Square	F	Significance

Strain	73934.94	35	2112.43	8.73	.0001
Error	28806.79	119	242.07		
Total	102741.73	154			

**Table 3:** LRS for regressed cortical and noncortical volume. The criteria LRS values for regressed cortical volume are 21.18 (highly significant at the genome-wide 0.001 level), 17.37 (significant at the genome-wide 0.05 level) and 9.91 (suggestive), following the conventions of Lander and Kruglyak [33]. The corresponding values for regressed noncortical brain volume are 20.90 (highly significant), 17.00 (significant) and 9.89 (suggestive). Genome-wide highly significant findings are marked by double asterisks, significant findings by a single asterisk. Chromosomal position is given in megabases.

**Chromosome**	**Marker**	**Position**	**LRS**
**Regressed Cortical Volume**			

11	D11Mit19	25.4	22.66**
11	S11Gnf020.100	21.0	17.45*
11	D11Mit135	33.7	16.39
11	D11Mit231	35.8	11.45
11	D11Mit51	36.7	10.14
16	D16Mit100	11.6	11.13
16	D16Mit146	23.5	10.18

**Regressed Noncortical Volume**			

19	D19Mit22	10.1	17.54*
19	D19Mit42	0.4	13.85
19	D19Mit127	12.1	12.39
11	D11Mit19	25.4	12.31
11	D11Mit231	35.8	10.38
